# Genetic characterization of highly pathogenic avian influenza A (H5N8) virus isolated from domestic geese in Iraq, 2018

**DOI:** 10.1186/s12917-021-02831-y

**Published:** 2021-03-19

**Authors:** Nahla Muhammad Saeed, Peshnyar Muhammad Atta Rashid, Hiewa Othman Dyary

**Affiliations:** 1 College of Veterinary Medicine, University of Sulaimani, Sulaymaniyah, Kurdistan Region Iraq; 2 Molecular Diagnostic Laboratory, Directorate of Veterinary Services, Sulaymaniyah, Kurdistan Region Iraq; 3 Kurdistan Institution for Strategic Studies and Scientific Research, Sulaymaniyah, Kurdistan Region Iraq

**Keywords:** Highly pathogenic avian influenza, H5N8, Iraqi Kurdistan Region

## Abstract

**Background:**

Influenza viruses are a continuous threat to avian and mammalian species, causing epidemics and pandemics. After the circulation of H5N1 in 2006, 2015, and 2016 in Iraq, an H5N8 influenza virus emerged in domestic geese in Sulaymaniyah Province, Iraq. This study analyzed the genetic characteristics of the Iraqi H5N8 viruses.

**Results:**

An HPAI virus subtype H5N8 was identified from domestic backyard geese in the Kurdistan Region, north Iraq. Phylogenic analyses of the hemagglutinin (HA) and neuraminidase (NA) genes indicated that Iraq H5N8 viruses belonged to clade 2.3.4.4 group B and clustered with isolates from Iran, Israel, and Belgium. Genetic analysis of the HA gene indicated molecular markers for avian-type receptors. Characterization of the NA gene showed that the virus had sensitive molecular markers for antiviral drugs.

**Conclusions:**

This is the first study ever on H5N8 in Iraq, and it is crucial to understand the epidemiology of the viruses in Iraq and the Middle East. The results suggest a possible role of migratory birds in the introduction of HPAI subtype H5N8 into Iraq.

## Background

Influenza A is a genus of the *Orthomyxoviridae* family that causes a highly infectious disease affecting poultry populations worldwide [1]. Avian influenza viruses (AIV) are divided into highly pathogenic avian influenza (HPAI), essentially a poultry disease, and low pathogenic avian influenza (LPAI) [2]. Viruses causing LPAI affect many wild bird species of *Anseriformes* and *Charadriiformes* [3, 4].

Strains of H5N8 HPAI were first identified in wild birds in 2010 and spread to domestic birds in China [5]. An epidemic of H5N8 HPAI in 2014/2015 resulted in the loss of more than 50 million birds in Asia, Europe, and North America [6]. In 2016/2017, another H5 HPAI epidemic spread from Asia and caused the largest recorded epidemic of domestic and wild birds in Europe [7].

Influenza A viruses have negative-sense RNA genomes composed of eight segments. Based on the combination of two surface glycoproteins, hemagglutinin (HA) and neuraminidase (NA), Influenza A viruses are divided into H1 to -16 HA and N1 to -9 NA subtypes [8]. HPAI viruses of H5 and H7 subtypes cause devastating losses in the poultry industry globally because of high mortality rates in infected flocks [9].

Avian influenza viruses evolve rapidly because of the high error rates produced by the viral RNA polymerase [10]. Since the first detection of HPAI subtype H5N1 in China in 1996, the virus’s HA gene has evolved into ten phylogenetic clades (0–9) [11]. Due to increased diversity, these were divided into suborder clades and subclades. The HA gene of H5 subtypes underwent reassortment with various neuraminidase subtypes (1–9) to form AI H5NX viruses [12].

The subtypes of H5N8 that belong to H5 clade 2.3.4.4 were first isolated from poultry farms in China in 2010 [13]. Outbreaks of H5N8 were then reported in South Korea in early 2014 in chickens and domestic ducks [14, 15]. By the end of 2014, H5N8 spread throughout Europe, North America, and East Asia [16–18].

The first report of HPAI subtype H5N1 in Iraq was from humans in Sulaymaniyah Governorate, Kurdistan region in 2006 [19]. In May 2015, an isolated H5N1 outbreak was detected in the backyard poultry in Sulaymaniyah Governorate, as was stated by the Ministry of Agriculture in the Kurdistan Region [20]. In 2016, the World Organization for Animal Health (OIE) released two reports in Iraq, indicating 17 outbreaks of H5N1 in broiler farms. On January 7, 2018, OIE declared the first H5N8 outbreaks in Iraq, followed by ten reports indicating 16 outbreaks in broiler chicken farms [21]. In 2019, OIE declared only one outbreak of H5N8 in April in the south of Iraq [22].

In 2018, H5N8 avian influenza was identified in backyard domestic geese in Sulaymaniyah Province. This is the first study analyzing the genetic characteristics of the Iraqi H5N8 viruses because it is the only H5N8 viral sequence available in GenBank databases. The phylogenetic analysis, focusing on HA and NA proteins, provided information to identify the closely-related viruses to understand the virus’s epidemiology in the area.

## Results

### Genetic analysis of HA and NA

**Fig. 1:**
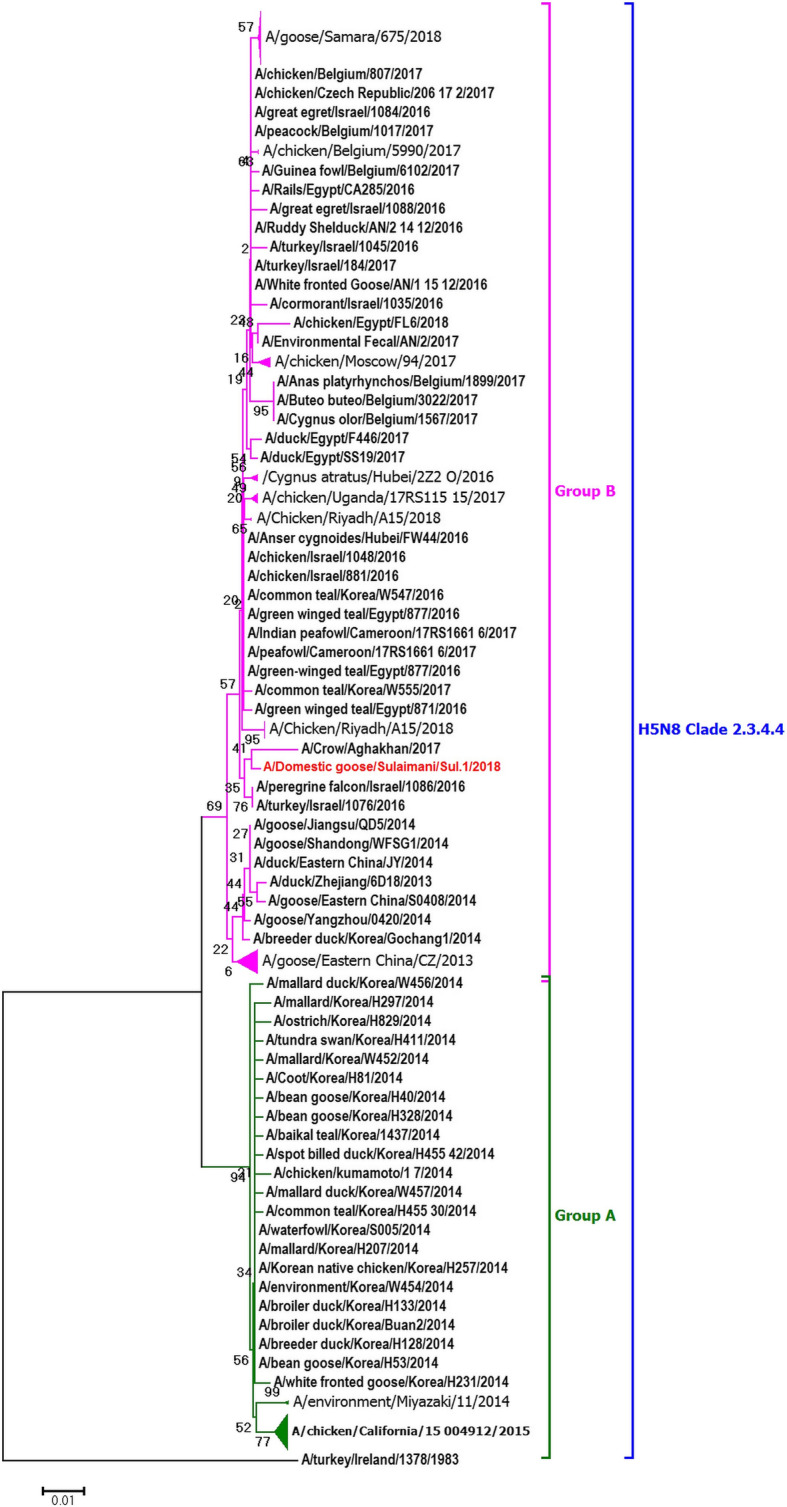
Phylogenetic tree of HA amino acid sequences estimated with the Neighbor-Joining algorithm using MEGA version 7. The topology was supported by bootstrap analysis with 1000 replicates. Iraq H5N8 viruses are depicted as red circles. It fell in clade 2.3.4.4 of group B

The phylogenetic tree’s topology based on the HA gene showed that the A/Domestic goose/Sulaimani/Sul.1/2018 viruses from Iraq belonged to clade 2.3.4.4 group B (Fig. 1). The phylogenetic tree revealed that A/Domestic goose/Sulaimani/Sul.1/2018was closely clustered with isolates of wild and domestic birds in Iran and Israel, namely A/Crow/Aghakhan/2017, A/peregrine falcon/Israel/1086/2016, and A/turkey/Israel/1076/2016. The phylogenic analysis of the partial NA gene of the Iraq H5N8 virus also showed that it belonged to group B, and it was clustered with viruses from wild birds in Belgium, namely A/Anas platyrhynchos/Belgium/1899/2017, A/Buteo buteo/Belgium/3022/2017, and A/Cygnusolor/Belgium/1567/2017.

The amino acid sequences of A/Domestic goose/Sulaimani/Sul.1/2018 virus showed that the virus had multibasic cleavage sites of HPAI in the motif PLREKRRKR.GLF, which is the molecular marker of HPAI. The receptor binding sites of the H5 contained H103, E186, N189, K192, K189, G221, Q222, R223, and G224 (H5 numbering). The HA gene was also characterized by having A133 and A156 amino acid residues. A/Domestic goose/Sulaimani/Sul.1/2018 had amino acids S94, V282, and I114, which is characteristic of 2.3.4.4 group B. Amino acid characterization of NA gene of A/Domestic goose/Sulaimani/Sul.1/2018 indicated that the gene possessed the amino acid residues V116, I117, R118, E119, Q136, V148, D151, R155, D198, I222, S246, H277, E276, R292, and N294 (N2 numbering).

## Discussion

In this study, HPAI (H5N8) virus, A/Domestic goose/Sulaimani/Sul.1/2018, was detected in geese in Sulaymaniyah province in Kurdistan Region, Iraq. Phylogenetic analysis reveals that the virus fell in group B in clade 2.3.4.4 H5N8. The phylogenetic tree’s topology based on the HA gene indicated that the Iraq virus clustered with viruses isolated from Iran and Israel in 2016–2017. The phylogenetic tree’s topology based on the NA gene showed that the Iraq virus clustered with viruses in Iran and Belgium. According to both HA and NA clusters, A/Domestic goose/Sulaimani/Sul.1/2018 has a common ancestor with A/Crow/Aghakhan/2017, isolated from the migratory hooded crow in a national park in Esfahan province of Iran [23].

Because Iraq and Iran are located in the path of Black Sea-Mediterranean flyways and West Asian-East African flyways of migratory birds [24], it was suggested that both A/Domestic goose/Sulaimani/Sul.1/2018 and A/Crow/Aghakhan/2017 might have originated from the same source of migratory birds. Furthermore, according to previous database research on the transmission of H5N1 in Iraq, poultry trading is more likely associated with avian influenza transmission [24]. Iraq shares a long international border with Iran, and legal and illegal commercial and poultry trades occur between the two countries. Therefore, we cannot exclude that A/Domestic goose/Sulaimani/Sul.1/2018 may have been transmitted from Iran. As there were no reported HPAI cases in Sulaimani province and nearby provinces, it is difficult to estimate that the infection originated from indirect contacts with domestic birds and broiler farm chickens in Sulaimani Province.

According to the World Organization for Animal Health (OIE) report, the first outbreak of H5N8 in Iraq was in January of 2018 [21]. However, the sequences of the viral HA and NA have not been identified previously. Hence, our study about the characterization of the Iraqi H5N8 in Greylag Geese is considered the first report of a fully characterized HPAI H5N8 subtype.

Unfortunately, few sequences of H5N8 were available in the GenBank databases from the Middle East, and only 34 H5N8 isolates were submitted to GISAID across the globe, which hindered the analysis of the avian influenza virus isolated in this study. On the other hand, the other six H5N8 segments were deposited much less in GenBank than HA and NA. Therefore, we depended only on the analysis of HA and NA in this study.

Multiple insertions of basic amino acids at the HA gene’s cleavage site were major determinants of the H5 virus pathogenicity [25]. The sequence of the HA gene of A/Domestic goose/Sulaimani/Sul.1/2018 showed that the virus possessed the molecular markers for HPAI, with a polybasic amino acid cleavage site motif, PLREKRRKR/GLF. The receptor-binding site curtails the host range of the influenza virus [26]. The single amino acid substitutions A132S, Q222 L, G224S, Q192H, Q192R, S223 N, and N220 K (H5 numbering) of HA protein have been reported to increase the affinity of avian influenza virus to from α-2,3 sialic acid (avian) to α-2,6 sialic acid (human)[27, 28].

The receptor-binding domains of A/Domestic goose/Sulaimani/Sul.1/2018 were H103, E186, N189, K192, K189, G221, Q222, R223, and G224, which revealed the preference for classic avian α-2,3 sialic acid specificity. In this study, the Iraq HPAI subtype H5N8 had two substitutions in HA at S133A and T156A, like most H5N8 subtypes. These substitutions increase the affinity for α2,6 sialic acid receptors in mammals [29, 30]. Despite that, according to WHO and OIE, there were no reported human infection cases with the H5N8 influenza virus so far [21, 31].

The susceptibility of avian influenza to antiviral drugs is associated with NA protein sequence characteristics [32]. Previous studies showed that molecular markers of resistance to zanamivir are V116A, R118K, E119G/A/D, Q136K, D151E, R152K, E277D, R292K, and oseltamivir resistance markers are I117V, E119V, D198N, H274Y, R292K, and N294S (N2 numbering) [33]. Analysis of the NA-deduced amino acid of Iraq H5N8 showed no markers for oseltamivir, zanamivir, and Peramivir. Therefore, A/Domestic goose/Sulaimani/Sul.1/2018 may be susceptible to antiviral drugs that act via NA inhibition.

## Conclusions

The genetic characteristics of the HA gene of the Iraq H5N8 virus revealed molecular markers for the avian type receptor. The phylogenic analysis showed that the Iraq H5N8 virus fell in clade 2.3.4.4 group B and clusters with some Middle East H5N8 viruses. Genetic characterization of NA showed susceptibility of the virus to antiviral drugs. There was not enough information in the major sequence databases about the H5N8 viral sequence in the Middle East, especially in Iraq, which negatively affected our avian influenza research. Further surveillance on full-genome analyses is needed to determine the main risk factors for HPAI H5N8 viruses in Iraq.

## Methods

### Outbreak history

Influenza infection was suspected in domestic graylag geese (*Anser anser*) raised in a privately-owned farm in Sulaymaniyah province, north Iraq, in November 2018. About 200 geese were raised in backyards in a populated area and were divided into several flocks. There were no other birds raised in the backyards. The clinical signs were loss of appetite, torticollis, convulsions, blindness, and death within one or two days after the onset of symptoms. The mortality rate was about 30 %, and avian influenza was suspected according to the clinical signs.

### RNA extraction

Tracheal mucus and lung tissue were collected from two 12-month-old geese and pooled together. Total RNA extraction was conducted using a total RNA extraction kit (GeNet Bio, South Korea), following the manufacturer’s instructions.

### Oligonucleotides and Reverse Transcription PCR (RT-PCR) amplification

The *M* gene was used first to diagnose Influenza virus A [34]. Sets of primers were designed and used to identify and sequence the H5N8 virus’s *HA* and *NA* genes (Table 1). Macrogen®, Korea, produced all the primers.

**Table 1:** List of primers and amplified genes

Primer name	sequences	Gene	Purpose	Amplicon	Reference	Reference strain
M30F2/08F	ATGAGYCTTYTAACCGAGGTCGAAACG	M	Detection of AIV type A	244	[34]	26–52
M264R3/08R	TGGACAAANCGTCTACGCTGCAG	M	Detection of AIV type A		[34]	267 − 245
HA5-1 F	AAAGTGATCAGATYTGCATTG	HA	Sequencing	420	[20]	44–64
HA5-1R	TGGTATGGRCATGCTGAGCTCA	HA	Sequencing		[20]	440–461
H5-2 F	TCATTTTGAGAAGATTCTGATCATCC	HA	Sequencing	754	This study	375–400
H5-2R	CCCCTGCTCATTGCTATGGT	HA	Sequencing		This study	1128 − 1109
H5-3 F	GGCAACGTGGAAGAATGGAC	HA	Sequencing	744	This study	617–636
H5-3R	ACTCGAAACAACCGTTACCC	HA	Sequencing		[34]	1360 − 1341
H5-4 F	CATCCACCCTCTCACCATCG	HA	Sequencing		[20]	927–968
H5-4R	GCGATCCATTGGAGCACATC	HA	Sequencing		[20]	1681 − 1662
NA8-1 F	AATAATGACCGTTGGCTCCA	NA	Detection & sequencing	616	This study	18–37
NA8-1R	AGTAGGCACCCCTCCGTAAT	NA			This study	633 − 614
NA8-2 F	AAGTGGATGGCGATTGGTGT	NA	Sequencing	403	This study	567–586
NA8-2R	TGGGCAACCCTGCACATAAA	NA	Sequencing		This study	969 − 950

A one-step RT-PCR was used for viral nucleic acid amplification. The *M, HA*, and *NA* genes were amplified separately using SuPrimeScript RT-PCR Premix manufactured by GeNet Bio, South Korea. The reaction was carried out in 0.2 mL PCR tubes containing 10 µL master mix, 4 µL RNA, 1 µL (10 pmol) of each primer (forward and reverse). Four microliters of diethylpyrocarbonate-treated water were then added to a final volume of 20 µL (Table 1).

The thermal cycler was initially set at 50 °C for 30 min. The PCR started with denaturation at 95 °C for 10 min. After that, 40 denaturation (95 °C for 30 s), annealing, and extension (72 °C for 50 s) cycles were run. A final extension at 72 °C for 4 min was also included. The *M* gene annealing temperature was set at 52 °C for 30 s. Meanwhile, the HA and NA primers’ sequencing temperature was set at 57 °C for 50 s.

PCR products were analyzed by loading 7 µL on 1 % agarose gel in 1× Tris/Borate/EDTA (TBE) buffer. The gel was stained with 10 µL safe dye. Electrophoresis was run at 130 volts for 1 h on the Safe-Blue Illuminator/Electrophoresis System. The amplicon of PCR products (Table 1) was analyzed according to the 100 bp DNA ladder’s migration pattern.

### Sequencing and phylogenic analysis

The PCR products were sequenced in the Macrogen Sequencing Facility in South Korea. Each nucleotide’s identity was verified by sequencing from both ends via reverse and forward primers. The coding sequences were uploaded to the GenBank Influenza virus database. The virus was named based on the WHO system of influenza viruses nomenclature by A/Domestic goose/Sulaimani/Sul.1/2018 [35] and received the accession numbers MK757595 and MK757597 for HA and NA, respectively.

**Fig. 2:**
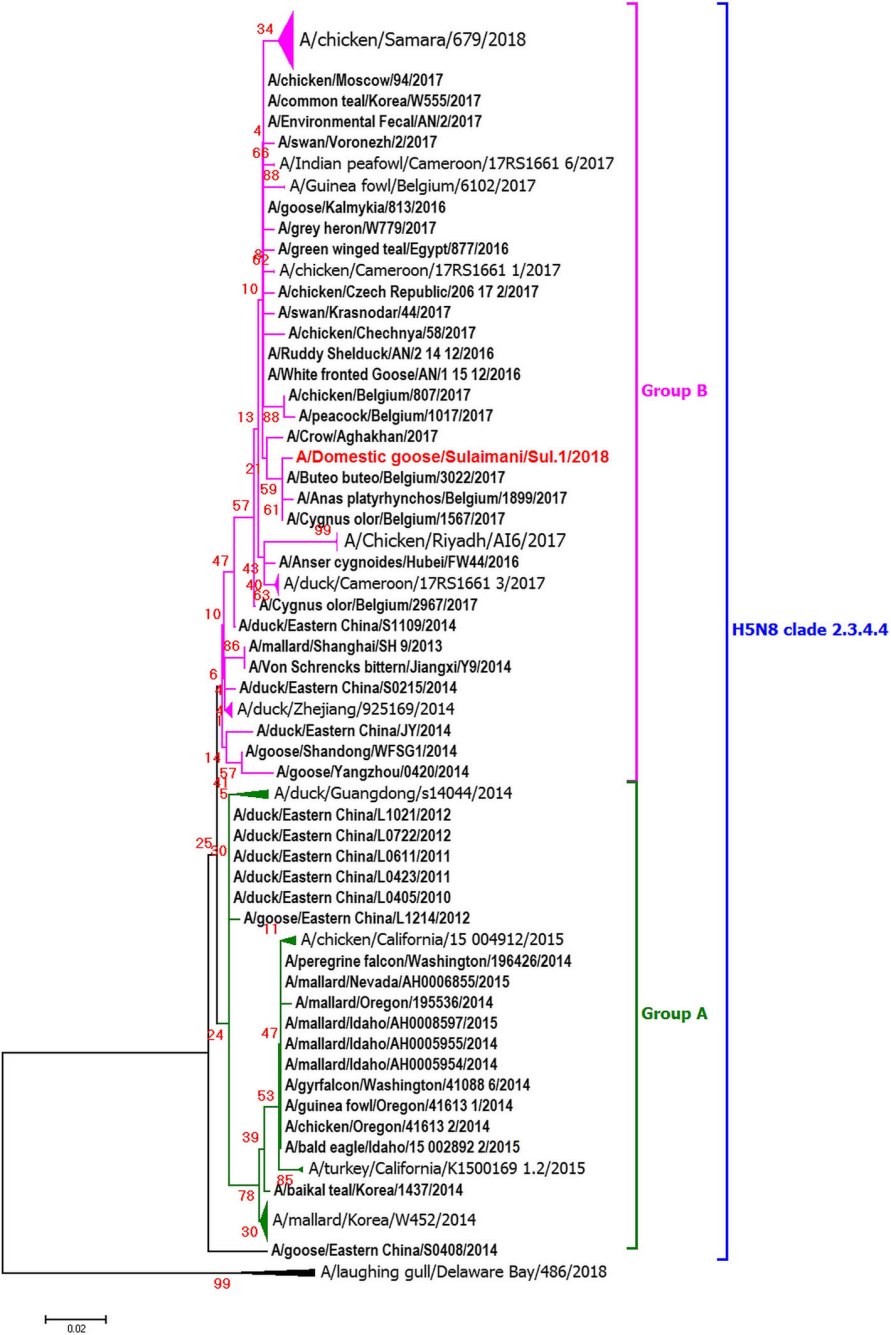
Phylogenetic tree of NA amino acid sequences estimated with the Neighbor-Joining algorithm using MEGA version 7. The topology was supported by bootstrap analysis with 1000 replicates. Iraq H5N8 viruses are depicted as red circles. It fell in clade 2.3.4.4 of group B

The partial HA (1065 bp) and NA (885 bp) genes were used to construct a phylogenic tree of 175 H5N8 virus strains (Figs. 1 and 2). The sequences were obtained from Global Initiative on Sharing Avian Influenza Data (GISAID) and the National Institute of Allergy and Infectious Diseases (NIAID) Influenza Research Database (IRD) through the website [36]. Multiple sequence alignments were conducted with the Clustal W method [37]. MEGA 7 was used to perform phylogenetic analysis with Neighbor-Joining. A/turkey/Ireland/1378/1983 virus was used as an outgroup to root the trees. The bootstrap values were decided from 1000 original data replicates.

### Ethics approval and consent to participate

The study was conducted following the Animal Care and Use Committee (ACUC) ethical guidelines at the College of Veterinary Medicine, University of Sulaimani. The ACUC approved the study (approval number 19/5), and verbal consent to collect samples from the infected animals was provided by the farm owner. After confirmation that influenza infection has spread on the farm, the animals were humanely euthanized by an overdose injection of sodium pentobarbital and disposed of by burying in a permitted landfill.

## Data Availability

The coding sequences of the HA and NA gene were submitted to the GenBank Influenza virus database. The virus was named by A/Goose/Iraq/Sul.1/2018. The accession numbers of the HA and NA genes are MK757595 and MK757597, respectively.

## Abbreviations

ACUC: Animal Care and Use Committee
AIV: Avian Influenza Virus
DEPC: Diethyl pyrocarbonate
EDTA: ethylenediaminetetraacetic acid
GISAID: Global Initiative on Sharing Avian Influenza Data
HA: Hemagglutinin
HPAI: Highly Pathogenic Avian Influenza
IRD: Influenza Research Database
LPAI: Low Pathogenic Avian Influenza
NA: Neuraminidase
NIAID: National Institute of Allergy and Infectious Diseases
OIE: World Organization for Animal Health
TBE: Tris/Borate/EDTA
WHO: World Health Organization
